# The epigenetic regulatory network of long noncoding RNAs in hepatocellular carcinoma

**DOI:** 10.1016/j.gendis.2025.101534

**Published:** 2025-01-20

**Authors:** Zhaoqi Shi, Shengxi Jin, Xiaolong Liu, Mengting Jiang, Yifeng Fang, Parikshit Asutosh Khadaroo, Hui Lin, Xiaoxiao Fan

**Affiliations:** a Department of General Surgery, Sir Run Run Shaw Hospital, School of Medicine, Zhejiang University, Hangzhou, Zhejiang 310016, China; b Neuropsychiatry & ECT Department, Royal Melbourne Hospital, Grattan Street Parkville, Melbourne, VIC 3052, Australia; c College of Biomedical Engineering and Instrument Science, Zhejiang University, Hangzhou, Zhejiang 310027, China

**Keywords:** DNA methylation, Epigenetics, Hepatocellular carcinoma, Histone modification, Long noncoding RNA, MicroRNA, RNA methylation

## Abstract

Long noncoding RNAs (lncRNAs) are endogenous noncoding RNAs exceeding 200 bases in length that are prevalent in malignant tumors and are closely associated with the onset and progression of hepatocellular carcinoma. The synthesis of lncRNAs exhibits similarities to that of protein-coding transcripts, which is regulated by epigenetic modifications. Recent research has highlighted the significant regulatory role of epigenetic modifications in the transcription of lncRNA genes in hepatocellular carcinoma. This review outlines the impact of epigenetic modifications, including DNA methylation, histone modification (methylation and acetylation), RNA modification, and microRNAs on the transcription of lncRNA genes in hepatocellular carcinoma and delves into the underlying mechanisms by summarizing how these lncRNA genes contribute to the development and progression of hepatocellular carcinoma.

## Introduction

Long noncoding RNAs (lncRNAs) are noncoding RNA molecules exceeding 200 nucleotides in length that are characterized by limited or absent protein-coding capacity.^1^ They can engage in various biological processes, including the regulation of gene expression, chromatin remodeling, transcription, post-transcriptional regulation, splicing, and translation, through their interactions with DNA, proteins, or other RNA molecules^2^ lncRNAs can be categorized into five distinct groups based on the genomic locations of their coding sequences: intergenic, intronic, bidirectional, sense, and antisense lncRNAs.^3^ These genes generally display transcription patterns that are comparable to those of protein-coding genes.^4^ Typically, the promoter region of lncRNA gene sequences exhibits active chromatin markers, such as H3K27 acetylation, as well as H3K4 dimethylation or trimethylation.^5^^,^^6^ These markers facilitate the binding and activation of RNA polymerase II, initiating lncRNA transcription and yielding multiple transcripts.^7^^,^^8^ Following transcription, most nascent lncRNA transcripts undergo processing akin to canonical RNA processing, involving the addition of a 5′-terminal methyl guanosine cap, 3′-terminal polyadenylation for poly-A tail formation, and splicing to excise introns.^9^ Some lncRNA gene sequences even share promoters with protein-coding genes, and during embryonic development, their transcriptional processes are coordinated with those of coding genes.^10^ Such similarity in biosynthesis processes between lncRNAs and protein-coding transcripts suggests the existence of analogous transcriptional regulation patterns in lncRNA transcription as in coding genes. Previous research has indicated that transcription factors, DNA methylation, and histone modifications are capable of regulating lncRNA expression at the transcriptional level, while RNA modifications, RNA-binding proteins, and microRNAs (miRNAs) can influence lncRNA expression at the post-transcriptional level.^11^^,^^12^

lncRNAs play a pivotal role in the complex biological landscape of cancer, influencing tumorigenesis and cancer progression through various mechanisms. lncRNAs contribute to cancer phenotypes such as uncontrolled cell proliferation, survival, migration, and genomic instability.^13^ In the context of cancer metabolism, lncRNAs have been shown to regulate glucose and lipid metabolism in tumor cells, which is critical for sustaining the energy demands of cancer cells.^14^ Furthermore, lncRNAs are implicated in the regulation of the tumor microenvironment and immune response, offering potential targets for immunotherapy.^15^ Understanding the multifaceted roles of lncRNAs in cancer provides valuable insights for the development of novel therapeutic interventions and diagnostic tools.

Hepatocellular carcinoma (HCC) is a malignant neoplasm characterized by high morbidity and mortality. Recent data from the World Health Organization in 2020 revealed that HCC was the sixth most diagnosed cancer and the third leading cause of cancer-related deaths globally.^16^ Researchers have long sought to elucidate the pathogenesis of HCC, with the prevailing theory suggesting that HCC arises from the accumulation of genetic mutations and epigenetic alterations in proto-oncogenes and driver genes, resulting in molecular heterogeneity.^17^^,^^18^ Epigenetic modifications, frequently observed in HCC, can induce genomic instability, thereby augmenting the likelihood of gene mutations.^19^, ^20^, ^21^ HCC is distinguished by a unique microenvironment that includes viral infections, hypoxia, and immunosuppression, which significantly alter the epigenetic landscape of the liver and contribute to the development of HCC.^22^ lncRNAs are crucial in this context as they interact with the epigenome and modulate the expression of genes that are critical in the response to these specific microenvironmental stresses.^23^^,^^24^ Emerging research indicates that lncRNAs play a crucial epigenetic regulatory role in the initiation and progression of HCC, influencing various biological processes, such as cell proliferation, apoptosis, metastasis, invasion, angiogenesis, immune evasion, alterations in the tumor microenvironment, and the development of drug resistance.^25^, ^26^, ^27^ Furthermore, the expression levels of lncRNAs in HCC have shown promise as diagnostic markers^28^^,^^29^ and potential therapeutic targets^30^, ^31^, ^32^ for this disease.

**Figure 1:**
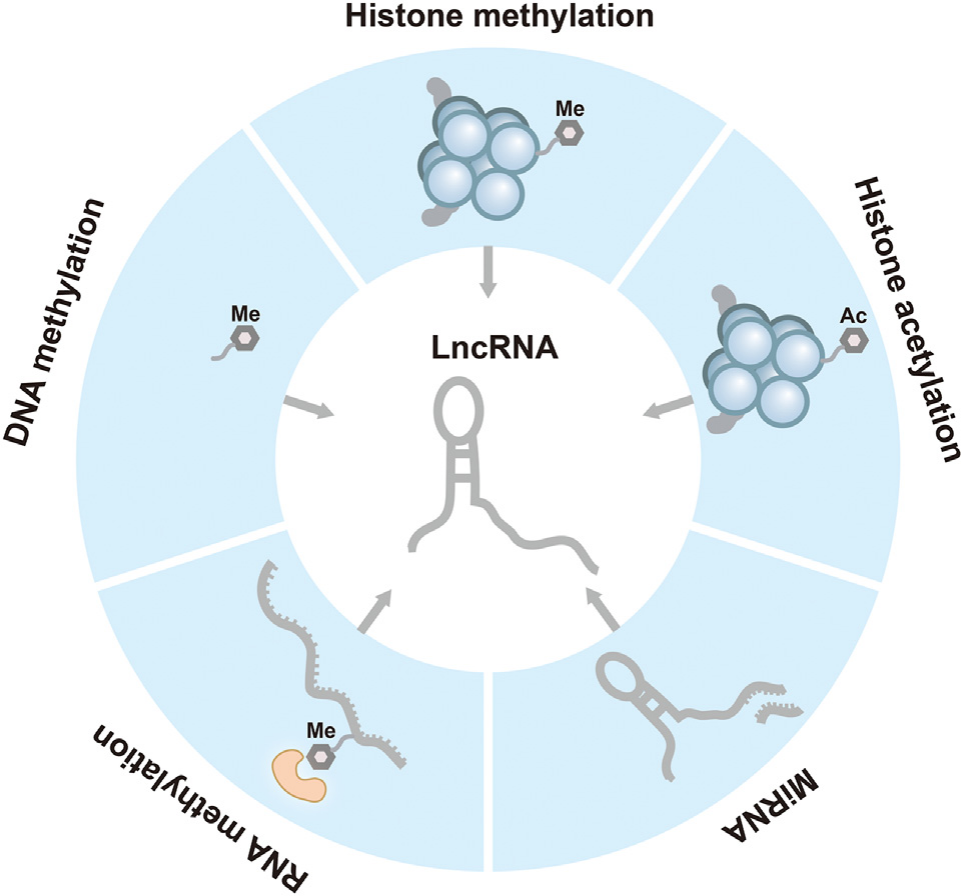
The epigenetic regulatory network of lncRNAs in hepatocellular carcinoma. The regulation of lncRNA expression in hepatocellular carcinoma involves epigenetic mechanisms such as DNA methylation, histone methylation, histone acetylation, RNA methylation, and miRNA modification.

**Figure 2:**
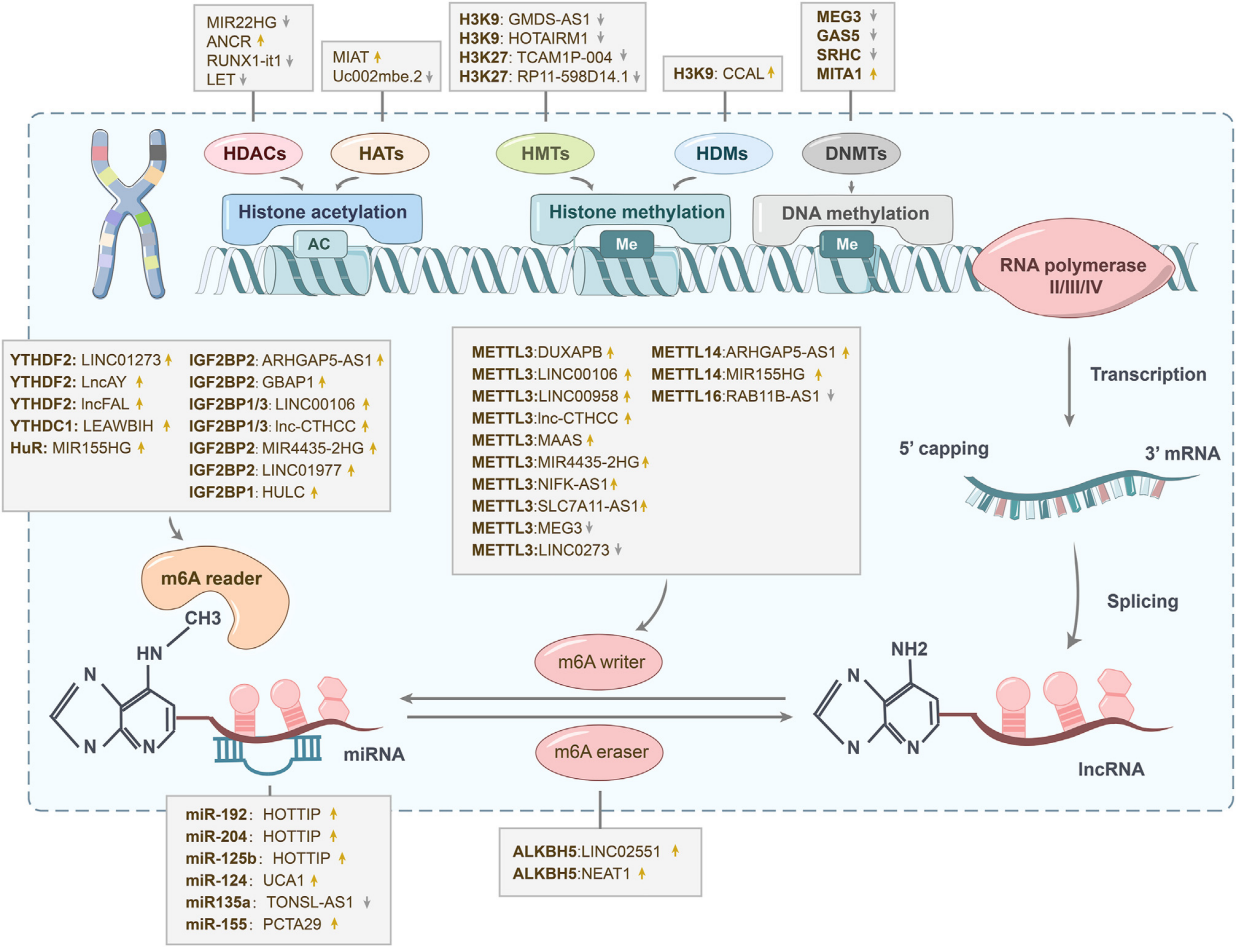
Overview of lncRNAs regulated by epigenetic modifications in hepatocellular carcinoma. The transcription of lncRNA genes in hepatocellular carcinoma can be regulated by epigenetic modifications such as histone methylation, histone acetylation, and DNA methylation. After transcription, lncRNA gene transcripts are further regulated at the post-transcriptional level by RNA methylation and miRNA binding. HDACs, histone deacetylases. HATs, acetyltransferases. HMTs, histone methyltransferases. HDMs, histone demethylases. DNMTs, DNA methyltransferases.

Presently, literature reviews focusing on lncRNAs in the context of HCC predominantly highlight their biological functions, downstream molecular mechanisms, and clinical implications while paying limited attention to the upstream transcriptional regulation of lncRNAs in HCC. This review aims to offer a comprehensive overview of the transcriptional regulatory mechanisms governing lncRNA expression in HCC, with a specific emphasis on five epigenetic aspects (Fig. 1). By elucidating the significance of epigenetic regulation in the transcriptional processes of lncRNAs in HCC, this review endeavors to advance comprehension in this field (Fig. 2).

## DNA methylation in the regulation of lncRNA expression

DNA methylation plays a crucial role in gene transcription regulation by inducing alterations in chromatin conformation or modifying the pattern of interaction between DNA and proteins without affecting the gene sequence.^33^ The process of DNA methylation necessitates the involvement of DNA methyltransferases, including the DNMT1 and DNMT3 families.^34^ The DNMT3 family comprises DNMT3A, DNMT3B, and DNMT3L, with DNMT3A and DNMT3B exhibiting methyltransferase activity responsible for *de novo* methylation,^35^ while DNMT3L, lacking methyltransferase activity,^36^ is involved in the assembly of DNA methylated polymers and the regulation of DNMT3A/3B catalytic activity.^37^^,^^38^ DNMT1 primarily participates in the methylation of newly synthesized DNA strands during DNA replication to maintain the accuracy of methylation during the replication process.^39^

lncRNAs exhibit a biosynthetic mechanism akin to that of protein-coding transcripts, underscoring the regulatory significance of DNA methylation in the promoter region of their coding sequence during lncRNA transcription. A common approach to investigating the regulation of lncRNA genes by DNA methylation in tumors involves the assessment of the methylation and expression levels of lncRNA genes through the utilization of methylation chips and RNA sequencing. Bioinformatics analysis has been employed to explore the impact of global methylation alterations on the expression of lncRNA genes. To date, two studies have utilized a similar methodology to examine genome-wide methylation and expression patterns of lncRNA genes in HCC while also evaluating the regulatory influence of methylation on lncRNA gene expression.^40^^,^^41^ In the first investigation, the researcher initially employed RNA sequencing to evaluate the expression of lncRNA genes in 20 HCC cases and corresponding adjacent tissues, followed by differential expression analysis, which identified 848 differentially expressed lncRNA genes. Subsequent analysis involved the assessment of the methylation levels of these 848 lncRNA genes and the examination of the relationship between their methylation levels and expression levels. Ultimately, 93 lncRNA genes exhibiting a significant negative correlation between methylation levels and expression levels were identified, with a Pearson correlation coefficient of less than −0.3.^40^ The second study utilized the TCGA database to perform a comprehensive omics analysis on the methylation patterns of the promoter regions of lncRNA genes. The authors initially identified 180 differentially expressed lncRNAs in HCC tissues compared with adjacent normal tissues through expression analysis. Subsequently, they performed linear model fitting using the expression and methylation data of these lncRNAs, calculated *p*-values, and applied false discovery rate adjustment. This process led to the identification of 41 lncRNAs that exhibited differential expression between HCC and normal tissues, with their expression levels significantly correlated with methylation levels, using a cutoff point of 0.01.^41^ Regrettably, the correlation observed between the methylation of lncRNA genes and their expression in the aforementioned studies was limited to statistical significance. The authors did not conduct additional investigations to determine whether the transcription of these lncRNAs was regulated by the DNA methylation of their gene sequences.

In addition to methylomic analysis, several studies have documented the role of DNA methylation in the transcriptional regulation of specific lncRNA genes in HCC. For instance, Braconi et al reported that the lncRNA MEG3 (maternally expressed 3) displays heightened promoter region methylation levels and reduced expression in HCC. Treatment of HCC cell lines with decitabine or silencing of DNMT1/3b led to substantial up-regulation of MEG3 expression. This up-regulation of MEG3 expression was found to enhance apoptosis and impede the proliferation of HCC cells.^42^ Furthermore, Zheng et al reported that the lncRNA SRHC was characterized by hyperexpression in both HCC tissues and cell lines. Methylation-sequencing analysis indicated that the promoter region of SRHC contains a CpG-rich island, which is hypermethylated in HCC cells. Subsequent demethylation experiments on SMMC-7721 and Hep3B cell lines revealed significant up-regulation of SRHC expression following demethylation treatment compared with the mock control.^43^ Another intriguing study explored the connections among a 5-base pair indel polymorphism (rs145204276) in the lncRNA GAS5 (growth arrest-specific 5) promoter region, the methylation level of this promoter, and the expression level of GAS5 in HCC.^44^ Researchers discovered a strong association between the deletion allele and increased levels of GAS5 expression, as well as heightened methylation of a neighboring CpG site within the promoter region of GAS5.^44^ Deletion of rs145204276 resulted in changes in both GAS5 expression levels and the methylation status of a specific CpG site within its promoter region. However, a definitive causal relationship between these alterations has not been conclusively established.

In addition to promoter methylation, gene body methylation can influence gene transcription.^45^^,^^46^ Notably, the modulation of lncRNA transcription through gene body DNA methylation has been observed in patients with HCC. Ma et al identified a lncRNA named metabolically induced tumor activator 1 (MITA1), which is markedly up-regulated in HCC cells by serum starvation. Through examination of the genetic sequence of MITA1, researchers identified a potential CpG-rich region located in the second intron of the MITA1 gene. Subsequent analysis using bisulfite conversion revealed a notable increase in DNA methylation levels within this CpG island of MITA1 under conditions of glucose deprivation. Furthermore, exposure to the methyltransferase inhibitor decitabine or genetic knockout of Dnmt3B resulted in a significant reduction in MITA1 expression induced by glucose starvation.^47^ The findings of this study collectively indicated that a lack of energy in HCC cells may lead to heightened methylation within the CpG island of the MITA1 gene body. This process further facilitates the up-regulation of MITA1 expression, thereby enhancing the migration and invasion capabilities of HCC cells.

## Histone modification in the regulation of lncRNA expression

**Figure 3:**
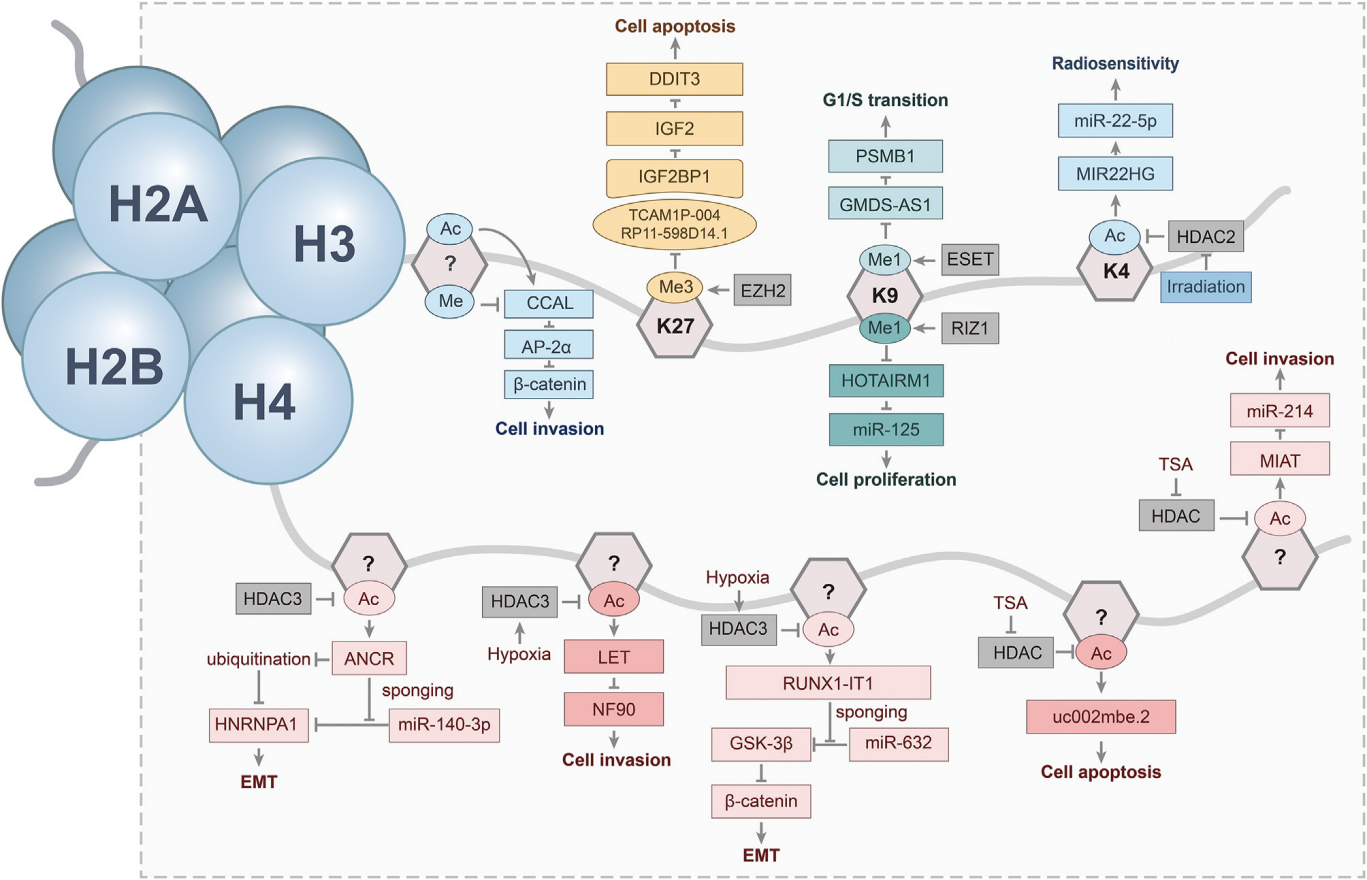
Histone modification plays a crucial role in the transcriptional regulation of lncRNAs in hepatocellular carcinoma. The histone modifications responsible for regulating lncRNA transcription in hepatocellular carcinoma predominantly target the H3 and H4 subunits. The identified regulatory sites include H3K9 monomethylation, H3K27 trimethylation, and H3K4 acetylation. While numerous other lncRNAs are subject to transcriptional regulation through histone modifications, the precise regulatory sites involved remain to be fully elucidated.

The nucleosome represents the fundamental structural component of the chromosome, consisting of a histone octamer comprising two sets of each of the four histone subunits, H2B, H2A, H3, and H4, around which DNA is damaged. Notably, each histone subunit contains an N-terminal tail, which serves as the location for histone modification on chromatin.^39^ Histone modification refers to the modification of amino acids located in the N-terminal tail of histone subunits through covalent bonding, including modifications such as methylation, acetylation, phosphorylation, and ubiquitination.^48^ Histone modifications can modulate gene transcription through alterations in chromatin structure compactness, thereby influencing the identification and binding of transcription factors.^49^^,^^50^ Histone modification is a prevalent epigenetic regulatory process that plays a crucial role in the initiation and progression of numerous types of tumors.^51^ Recent studies have indicated that histone modifications, mainly methylation and acetylation, play a significant role in the epigenetic regulation of lncRNA gene sequence transcription in HCC (Fig. 3 and Table 1).

**Table 1:** Histone modifications regulate lncRNA expression in hepatocellular carcinoma.

lncRNA	Expression	Biological function	Histone modification	Modification site	Regulation
GMDS-AS1^60^	Up	Proliferation, invasion, metastasis	Methylation	H3K9	Down
HOTAIRM1^61^	Down	Proliferation, metastasis	Methylation	H3K9	Down
TCAM1P-004^66^	Down	Proliferation, metastasis	Methylation	H3K27	Down
RP11-598D14.1^66^	Down	Proliferation, metastasis	Methylation	H3K27	Down
CCAL^67^	Up	Proliferation, invasion, metastasis	Methylation	H3	Down
MIR22HG^73^	Down	Radio sensitivity	Acetylation	H3K9	Up
ANCR^74^	Up	Invasion, metastasis	Acetylation	H3/H4	Up
RUNX1-it1^76^	Down	Proliferation, invasion, metastasis	Acetylation		Up
MIAT^77^	Up	Proliferation, invasion	Acetylation	H3/H4	Up
Uc002mbe.2^78^	Down	Apoptosis	Acetylation		Up
LET^75^	Down	Invasion, metastasis	Acetylation		Up

### Histone methylation

Histone methylation is the enzymatic process by which methyl groups are transferred from S-adenosylmethionine to specific amino acid residues located in the N-terminal tail of histones. This biochemical process is facilitated by histone methyltransferases and histone demethylases.^52^ In histone modification, lysine and arginine residues are frequently targeted for methylation,^53^ with the impact on gene expression being contingent upon the specific residue undergoing modification, the extent and distribution of methylation, and the genomic environment in which methylation takes place.^54^

Histone H3 lysine 9 methylation (H3K9me) occurs at the lysine 9 residue of the H3 histone protein. H3K9 can exist in three distinct methylation states, namely, monomethylated (H3K9me1), dimethylated (H3K9me2), and trimethylated (H3K9me3).^55^ Typically, H3K9me2 and H3K9me3 are associated with heterochromatin formation, resulting in the inactivation of gene transcription.^56^^,^^57^ The presence of H3K9me1 is linked to the inhibition of gene transcription, although the precise underlying mechanism is not fully understood.^58^^,^^59^ In HCC, two lncRNA genes have been linked to H3K9 monomethylation within their promoter regions. As illustrated by Huang et al, the lncRNA GMDS-AS1 (antisense RNA 1) demonstrates reduced expression levels in HCC and exerts anti-cancer effects by inhibiting the proliferation, metastasis, and invasion of HCC cells. In HCC cells, an elevated expression level of H3K9me1 in the promoter region of GMDS-AS1 has been observed. The histone methyltransferase ESET (Erg-associated SET domain), which is responsible for catalyzing H3K9 methylation, plays a pivotal role in this mechanism. The knockout of ESET leads to a decrease in H3K9me1 expression, consequently enhancing the expression of GMDS-AS1.^60^ Similarly, the lncRNA HOTAIRM1 (HOX antisense intergenic RNA myeloid 1), which is also regulated by histone methylation in HCC, can inhibit cell proliferation and metastasis. Retinoblastoma protein-interacting zinc finger gene 1 (RIZ1) increases the expression level of the histone methylase H3K9me1, which binds to the promoter region of HOTAIRM1, thereby repressing its transcription and reversing its inhibitory effects on HCC cells.^61^

Polycomb repressive complex 2 (PRC2) is a complex composed of multiple proteins that facilitate the methylation of lysine 27 on histone H3 (H3K27me).^62^^,^^63^ Enhancer of zeste 2 polycomb repressive complex 2 subunit (EZH2) serves as a crucial catalytic component within PRC2. This enzyme plays a key role in the methylation of H3K27, leading to the establishment of a histone modification known as H3K27me3.^64^ This modification is associated with the repression of gene expression.^65^ Xu et al reported that the expression of the lncRNAs TCAM1P-004 and RP11-598D14.1 was down-regulated in HCC tissues, leading to the inhibition of cell proliferation and metastasis *in vitro*, as well as tumor formation *in vivo*. The transcription of these two lncRNAs is suppressed by H3K27 trimethylation, which is mediated by the histone methyltransferase EZH2.^66^ The lncRNA CCAL (colorectal cancer-associated lncRNA) is potentially regulated by histone methylation in HCC. CCAL was found to promote the proliferation, metastasis, and invasion of HCC cells by activating the Wnt/β-catenin pathway. Researchers revealed a notable reduction in histone H3 methylation levels within the CCAL promoter region in HCC tissues compared with normal liver tissues. Furthermore, a negative association was observed between the levels of histone H3 methylation and CCAL expression in 37 HCC samples.^67^

### Histone acetylation

Histone acetylation involves the attachment of acetyl groups to lysine residues on histone proteins through the enzymatic activity of histone acetyltransferases, while histone deacetylases (HDACs) are responsible for the removal of acetyl groups from lysine residues on histones.^68^, ^69^, ^70^ Histone acetylation leads to a decrease in the positive charge of histones, thereby diminishing their attraction to negatively charged DNA molecules. This process ultimately causes a loosening of the chromatin structure. A relaxed chromatin structure facilitates the binding of transcription regulators, thereby facilitating the promotion of gene expression.^71^ Histone acetylation plays a crucial role in numerous biological processes within HCC, including metastasis, angiogenesis, metabolism, apoptosis, the tumor microenvironment, and immune homeostasis.^72^ The transcription of lncRNA genes in HCC is also regulated by histone acetylation, with a predominant mechanism involving the deacetylation process mediated by HDACs. Nevertheless, the impact of histone acetyltransferases on lncRNA transcriptional regulation in this context remains unexplored in the current literature, and the precise loci of deacetylation modulation have yet to be precisely identified.

In HCC cells, the expression of the lncRNA MIR22HG (MIR22 host gene) is typically low. However, radiotherapy could suppress the activity of the histone deacetylase HDAC2, leading to increased H3K4 acetylation in the promoter region of MIR22HG. It facilitates the transcription of MIR22HG, which subsequently increases the expression of miR-22-5p and enhances the sensitivity of HCC cells to radiotherapy.^73^ This is the only study that has clarified the specific site of histone acetylation.

In addition, several studies have reported the regulatory effect of histone acetylation on the transcription of lncRNAs in HCC. Nevertheless, these studies have not pinpointed the exact acetylation site. They have only validated the association between the transcriptional activity of lncRNA genes and the global level of histone acetylation within the promoter region. These previous studies focused on histone deacetylase 3 (HDAC3), an enzyme known for its deacetylase activity. The lncRNA ANCR (Angelman syndrome chromosome region) promoted the metastatic and invasive capabilities of HCC cells by inducing epithelial–mesenchymal transition. Wen et al reported that the acetylation levels of H3/H4 histones in the ANCR promoter region were elevated in HCC tissues and cells, resulting in the up-regulation of ANCR expression. Furthermore, the transcription of ANCR could be further increased by interfering with the activity of HDAC3.^74^ Hypoxia can induce the up-regulation of HDAC3 expression in HCC cells, causing a decrease in histone acetylation and reducing the transcription of lncRNAs. lncRNA LET was down-regulated in HCC, and hypoxia-induced HDAC3 could inhibit lncRNA-LET transcription by reducing histone acetylation-mediated regulation of the lncRNA-LET promoter region. Decreased expression of lncRNA-LET plays a crucial role in stabilizing the nuclear factor 90 protein, ultimately enhancing hypoxia-induced cancer cell invasion.^75^ Another lncRNA, RUNX1-IT1 (intronic transcript 1), which is derived from intron 1 of RUNX1, can inhibit the Wnt/β-catenin signaling pathway through the RUNX1-IT1/miR-632/GSK-3β (glycogen synthase kinase 3 beta) cascade, thereby impeding the proliferation, cell cycle progression, metastasis, and invasion of HCC cells. Hypoxia-induced HDAC3 inhibited the transcription of RUNX1-IT1, while treatment with the HDAC inhibitor trichostatin A (TSA) reversed this down-regulation.^76^

Additionally, the lncRNA MIAT (myocardial infarction-associated transcript), which is highly expressed in HCC, could enhance the proliferation and invasion abilities of HCC cells. The histone acetylation levels of H3/H4 in the MIAT promoter region were significantly greater in HCC tissues than in normal liver tissues. Treatment of HCC cells with TSA further increased the transcription level of MIAT, indicating the potential regulation of MIAT transcription by histone acetylation in HCC.^77^ Conversely, Uc002mbe.2 was down-regulated in HCC cells and tissues. Treatment with TSA up-regulated the transcription of Uc002mbe.2 in HCC cells, thereby promoting the apoptosis of HCC cells. Knockdown of Uc002mbe.2 expression could counteract the pro-apoptotic effect of TSA on HCC cell apoptosis.^78^ These two studies focused on the impact of TSA on lncRNA transcription in HCC but did not delve into the precise regulatory mechanism involved. The specific HDACs responsible for histone deacetylase activity in lncRNA transcription by targeting particular histone lysine sites remain uncertain.

## RNA modification in the regulation of lncRNA expression

**Figure 4:**
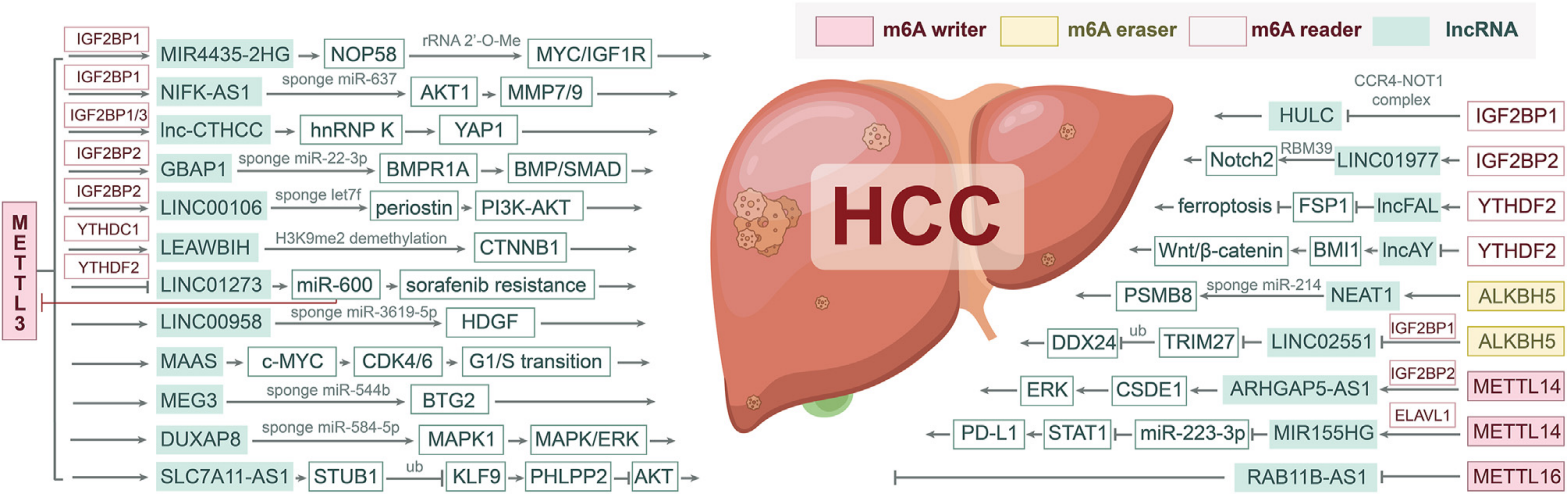
Post-transcriptional regulation of lncRNAs by m6A modifications in hepatocellular carcinoma. There are three types of molecules involved in m6A modification: m6A writers, m6A erasers, and m6A readers. m6A writers and erasers are responsible for adding and removing methyl groups to the N atom of adenosine 6 in RNA, while m6A readers primarily identify and recruit m6A writers or erasers, ensuring a dynamic balance between methylation and demethylation. Currently, most studies have explored the post-transcriptional regulatory effect of m6A writers, especially METTL3, on lncRNA expression in hepatocellular carcinoma, while less attention has been given to m6A erasers.

RNA modification is a form of post-transcriptional epigenetic regulation that is commonly observed in various types of RNA molecules, including messenger RNA (mRNA), transfer RNA (tRNA), ribosomal RNA (rRNA), miRNA, and lncRNA. These alterations impact the stability, subcellular distribution, transport, splicing, and translation of RNA molecules, consequently regulating their biological activities.^79^ To date, over 170 types of RNA modifications have been identified, with prevalent modes such as N6-methyladenine (m6A), 5-methylcytosine (m5C), pseudouracil, 5-hydroxymethylcytosine (hm5C), N1-methyladenine (m1A), and N7-methylguanosine (m7G).^80^ Notably, more than 10 types of RNA modifications have been reported to potentially modify lncRNA genes.^81^ The predominant RNA modifications identified in lncRNA genes associated with HCC are primarily m6A methylation,^82^^,^^83^ as outlined in Table 2. This specific modification involves the selective addition of methyl groups to the N atom of adenosine 6 in RNA, which is catalyzed by methyltransferase enzymes.^84^ m6A modification is a prevalent and distinctive internal modification in RNA that dynamically regulates RNA metabolism by influencing various effectors, impacting a wide array of physiological and pathological processes.^84^ In malignant tumors, m6A can act as both an oncogene and a tumor suppressor gene, affecting tumor development, metastasis, chemotherapy resistance, immune evasion, stem cell renewal, and tumor microenvironment regulation.^85^ The regulatory effectors of m6A modification can be categorized into m6A writers, m6A erasers, and m6A readers, each of which plays a distinct role in the modification process.^84^ The subsequent sections of this paper will delve into the regulatory impacts of RNA modification on lncRNA genes, focusing on these three aspects (Fig. 4).

**Table 2:** RNA modifications regulate lncRNA expression in hepatocellular carcinoma.

lncRNA	Expression	Biological function	Modification	Effector	Effector type	Regulation
ARHGAP5-AS1^101^	Hyper	Proliferation, migration, invasion	m6A	METTL14/IGF2BP2	m6A writer/reader	Up
DUXAP8^90^	Hyper	Proliferation, migration, invasion, drug resistance	m6A	METTL3	m6A writer	Up
GBAP1^89^	Hyper	Proliferation, migration	m6A	METTL3/IGF2BP2	m6A writer/reader	Up
HULC^126^	Hyper		m6A	IGF2BP1	m6A reader	Down
LEAWBIH^100^	Hyper	Proliferation, migration, invasion	m6A	METTL3/YTHDC1	m6A writer/reader	Up
LINC00106^91^	Hyper	Proliferation, stemness of cells	m6A	METTL3/IGF2BP1/3	m6A writer/reader	Up
LINC00958^92^	Hyper	Proliferation, migration, invasion	m6A	METTL3	m6A writer	Up
LINC01273^98^	Hyper	Drug resistance	m6A	METTL3/YTHDF2	m6A writer	Down
LINC01977^127^	Hyper	Proliferation, migration	m6A	IGF2BP2	m6A reader	Up
LINC02551^110^	Hyper	Proliferation, migration	m6A	ALKBH5/IGF2BP1	m6A eraser/reader	Down
lncAY^134^	Hyper		m6A	YTHDF2	m6A reader	Down
lnc-CTHCC^97^	Hyper	Proliferation, migration	m6A	METTL3/IGF2BP1/3	m6A writer/reader	Up
lncFAL^135^	Hyper	Decreased susceptibility to ferroptosis	m6A	YTHDF2	m6A reader	Up
MAAS^93^	Hyper	Proliferation	m6A	METTL3	m6A writer	Up
MEG3^94^	Hypo	Proliferation, migration, invasion	m6A	METTL3	m6A writer	Up
MIR155HG^103^	Hyper	Immune escape	m6A	METTL14/HuR	m6A writer/reader	Up
MIR4435-2HG^99^	Hyper	Proliferation, stemness of cells	m6A	METTL3/IGF2BP2	m6A writer/reader	Up
NEAT1^111^	Hyper	Proliferation, migration, apoptosis	m6A	ALKBH5	m6A eraser	Up
NIFK-AS1^95^	Hyper	Proliferation, migration, invasion, drug resistance	m6A	METTL3/IGF2BP1	m6A writer/reader	Up
RAB11B-AS1^106^	Hypo	Proliferation, migration, invasion, apoptosis	m6A	METTL16	m6A writer	Down
SLC7A11-AS1^96^	Hyper	Proliferation	m6A	METTL3	m6A writer	Up
H19^139^	Hyper	Proliferation, migration, invasion, angiogenesis	m5C	NSUN2	m5C writer	Up

### m6A writers

m6A writers include m6A methyltransferases, notably methyltransferase 3 (METTL3) and methyltransferase 14 (METTL14), along with the METTL3 adaptor WTAP (WT1 associated protein) and other associated proteins, such as KIAA14295, RBM15/15B (RNA binding motif protein 15/15B), and ZC3H137.^86^ Within the methyltransferase domain complex, METTL3 functions as the catalytic subunit, while METTL14 serves as an RNA-binding scaffold that activates and enhances the catalytic function of METTL3.^87^ Although WTAP lacks catalytic activity, it plays a role in recruiting METTL3 and METTL14, thereby modulating m6A levels during RNA transcription.^88^ Currently, studies on the regulation of lncRNA expression by m6A writers in HCC have focused mainly on METTL3, with limited exploration of the involvement of other members in this regulatory process (Table 2 and Fig. 4).

### METTL3

To determine the genome-wide regulatory impact of METTL3 on lncRNA expression in HCC, a screening strategy was used in a study carried out by Liu et al.^89^ The ectopic overexpression of METTL3 in HCC cell lines was subsequently confirmed by RNA sequencing to identify differentially expressed lncRNAs. Finally, nine up-regulated and five down-regulated lncRNAs were identified. The lncRNA GBAP1 (glucosylceramidase beta pseudogene 1) exhibited the highest level of up-regulation among all the lncRNAs and was chosen for further validation. Using the SRAMP algorithm, researchers identified four potential m6A modification sites within GBAP1 RNA. Disruption of these sites caused a significant reduction in the m6A content of GBAP1, leading to decreased expression levels. Additionally, immunoprecipitation experiments using an antibody specific for METTL3 revealed the association of METTL3 with GBAP1. Knockdown of METTL3 resulted in reduced m6A levels in GBAP1 and a subsequent decrease in its expression. Therefore, the authors concluded that METTL3 was involved in promoting GBAP1 expression in HCC through a m6A-dependent mechanism.

Other studies have employed a one-on-one strategy to investigate the regulatory impact of METTLE3 on specific lncRNAs. The primary approaches employed in these strategies involve assessing the relative expression levels of lncRNAs and their m6A modification status following METTL3 overexpression or knockdown. Additionally, in some instances, the stability of lncRNAs will also be evaluated using RNA stability assays following METTL3 interference. For example, the lncRNA DUXAP8 (double homeobox A pseudogene 8) is overexpressed in HCC and has been shown to enhance the proliferation, migration, invasion, and resistance to chemotherapy of HCC both *in vivo* and *in vitro*.^90^ Methylated RNA immunoprecipitation assay with quantitative PCR analysis revealed a notably greater level of the m6A modification of DUXAP8 in HCC cell lines (Huh7 and SK-Hep-1) than in normal hepatocyte lines. Knockdown of METTL3 led to a significant reduction in DUXAP8 expression, whereas overexpression of METTL3 increased DUXAP8 expression. Furthermore, the m6A enrichment of DUXAP8 in HCC cells decreased upon METTL3 silencing but increased with METTL3 overexpression. Silencing METTL3 in the presence of dactinomycin, an RNA synthesis inhibitor, reduced the stability of DUXAP8, while overexpressing METTL3 had the opposite effect. Overall, METTL3 plays a crucial role in enhancing the expression of DUXAP8 in HCC.^90^ Several other lncRNAs in HCC, including LINC00106,^91^ LINC0095,^92^ MAAS,^93^ MEG3,^94^ NIFK-AS1,^95^ SLC7A11-AS1,^96^ and CTHCC,^97^ are also regulated by METTL3. These studies employed comparable one-on-one strategies to validate those findings (Fig. 4).

Some studies have utilized novel approaches to confirm the regulatory impact of METTL3-mediated m6A modification on lncRNAs. A study conducted by Kong et al revealed that LINC01273 was markedly up-regulated in human HCC tissues and was linked to sorafenib resistance. Exogenous overexpression of METTL3 notably suppressed the expression of LINC01273 by diminishing its stability in HCC cells. By checking the entire length of LINC01273, a m6A site within the LINC01273 sequence was identified. Consequently, plasmids with mutations at this site were generated, and a luciferase reporter assay was employed to investigate whether this site served as a recognition site for METTL3. The results indicated that METTL3 was unable to bind effectively to the mutated LINC01273 sequence.^98^ Zhu et al employed a similar methodology to confirm the METTL3-mediated regulation of m6A on the lncRNA MIR4435-2HG in HCC. MIR4435-2HG significantly augmented the stem cell characteristics of HCC cells, thereby facilitating tumorigenesis both *in vitro* and *in vivo*. The methylated RNA immunoprecipitation assay revealed that the level of m6A-modified MIR4435-2HG was notably reduced upon interference with METTL3. Subsequent quantitative PCR analysis revealed that the suppression of METTL3 resulted in decreased expression of MIR4435-2HG in HCC cells. Furthermore, mutation of the m6A modification site in the MIR4435-2HG sequence abolished the increase in luciferase activity induced by METTL3.^99^ In addition to single-site mutations, some researchers have used single-base elongation- and ligation-based PCR amplification methods (SELECT) to assess the extent of m6A modification at specific sites. For example, a previous study indicated that m6A modification occurred at residues 2095 and 2679 of the lncRNA LEAWBIH (long non-coding RNA epigenetically activating Wnt/β-catenin signaling in HCC) in SK-HEP-1, HuH-7, and Hep3B cells. Moreover, the levels of m6A modification at both the 2095 and 2679 sites increased following the overexpression of METTL3. RNA stability experiments demonstrated that the overexpression of METTL3 led to enhanced stability of the LEAWBIH transcript.^100^ All these studies provided more direct evidence about the regulatory impact of METTL3 on lncRNA expression in HCC.

### METTL14

METTL14, another key component of the methyltransferase domain complex, is implicated in the m6A modification of lncRNAs in HCC (Fig. 4). Specifically, METTL14 regulated the transcription of the lncRNA ARHGAP5-AS1 in HCC, where ARHGAP5-AS1 acted as an oncogene and was highly expressed. Knocking out METTL14 using siRNA resulted in decreased levels of m6A modification and decreased ARHGAP5-AS1 expression.^101^ Additionally, recent studies have established a connection between the development of HCC and the presence of lipopolysaccharides in the intestinal flora.^102^ Lipopolysaccharide could increase the expression of METTL14, leading to elevated m6A methylation of the lncRNA MIR155HG and subsequent up-regulation of its expression. This process influences the expression of programmed death-1 (PD-1) in HCC and plays a significant role in immune regulation within HCC.^103^

### METTL16

METTL16, an additional m6A methyltransferase, is capable of independently catalyzing m6A methylation without the need to form complexes.^104^^,^^105^ METTL16 is also implicated in the transcriptional regulation of lncRNAs in HCC (Fig. 4). METTL16 could trigger m6A modification of the lncRNA RAB11B-AS1 by directly binding to RAB11B-AS1, resulting in decreased stability of the RAB11B-AS1 transcript and subsequent down-regulation of RAB11B-AS1. The functional role of RAB11B-AS1 *in vivo* involves inhibiting the proliferation, migration, and invasion of HCC cells and promoting the apoptosis of HCC cells, thereby impeding the progression of HCC.^106^

### m6A erasers

m6A erasers, which are m6A demethylases such as FTO (fat mass and obesity-associated) and ALKBH5 (AlkB homolog 5), are responsible for the removal of methyl groups at m6A sites from RNA molecules.^107^^,^^108^ FTO and ALKBH5 exhibit distinct demethylation mechanisms, with FTO converting m6A to N6-hydroxymethyladenine and N(6)-formyl adenosine before ultimately producing adenosine for demethylation,^109^ while ALKBH5 directly demethylates m6A to adenosine.^107^ Research on the regulation of lncRNAs by m6A erasers in HCC is limited (Fig. 4). Zhang et al reported that m6A methylation enhanced the stability of the lncRNA LINC02551 in HCC cells, leading to increased expression and promoting the proliferation, metastasis, and invasion of HCC cells. ALKBH5-mediated m6A demethylation could counteract this process, reducing LINC02551 expression and inhibiting its oncogenic effects.^110^ The other lncRNA affected by ALKBH5 in HCC is nuclear paraspeckle assembly transcript 1 (NEAT1), and ALKBH5 up-regulated NEAT1 expression by reducing its m6A levels. Increased NEAT1 expression in SMMC-7721 and Huh7 cells was associated with enhanced proliferation and migration of HCC cells, as well as the induction of apoptosis. NEAT1 functions by competitively binding to miR-214 and preventing its interaction with the target gene PSMB8 (proteasome 20S subunit beta 8).^111^

### m6A readers

m6A readers, also known as m6A methylation-binding proteins or recognition proteins, play crucial roles in identifying and recruiting m6A methyltransferases or demethylases to mRNA molecules, maintaining a dynamic balance between methylation and demethylation.^112^ This process helps regulate various biological functions of mRNAs, including metabolism, splicing, nucleation transport, translation, and stability.^113^ The m6A reader is composed of a group of proteins including five proteins with a YTH domain (YTHDF1, YTHDF2, YTHDF3, YTHDC1, and YTHDC2),^114^ three insulin-like growth factor 2 mRNA binding proteins (IGF2BP1, IGF2BP2, IGF2BP3),^115^ three heterogeneous nuclear ribonucleoproteins (hnRNPC, hnRNPG, hnRNPA2B1),^116^, ^117^, ^118^ and human antigen R (HuR).^119^^,^^120^

### IGF2BP family

The IGF2BP family comprises three members, namely, IGF2BP1, IGF2BP2, and IGF2BP3, which possess six RNA-binding domains. These include four C-terminal KH domains that facilitate RNA binding and two N-terminal RNA recognition domains that enhance RNA complex stability.^121^ The primary function of the IGF2BP family in m6A modification is to increase RNA molecule stability after modification.^122^ While the expression of the IGF2BP family is typically low in normal tissues, it is notably up-regulated in various malignant tumors and is closely correlated with the prognosis of patients with these cancers.^123^^,^^124^

In HCC, IGF2BP1 plays a synergistic role in the regulation of MIR4435-2HG,^99^ NIFK-AS1,^95^ and CTHCC^97^ via METTL3-mediated m6A modification (Fig. 4). RNA immunoprecipitation assays showed that IGF2BP1 could specifically bind to these three lncRNAs. Knockdown of IGF2BP1 decreased the expression of these three lncRNAs and attenuated the stability of the lncRNA CTHCC. Dual-luciferase assays revealed that mutations in the m6A modification site of the MIR4435-2HG sequence inhibited the binding of IGF2BP1 to MIR4435-2HG. IGF2BP1 is also involved in the regulation of LINC02551 by ALKBH5-mediated m6A modification in HCC.^110^ As mentioned above, ALKBH5 promoted the decay of LINC02551 in a m6A-dependent manner. The introduction of IGP2BP1 could stabilize LINC02551 and effectively inhibit the degradation caused by ALKBH5.^110^ In addition to stabilizing lncRNAs after m6A modification, IGF2BP1 can also facilitate lncRNA degradation. In HCC, the up-regulation of the lncRNA HULC (hepatocellular carcinoma up-regulated long noncoding RNA) was associated with the depletion of IGF2BP1, which recognizes m6A-modified HULC molecules and recruits the CCR4-NOT complex to initiate HULC degradation. The CCR4-NOT complex plays a crucial role as a deadenylase enzyme in the cytoplasm and is responsible for the shortening of poly(A) tails and initiating the degradation of various RNAs through a 3′-5′ decay mechanism.^125^ Consequently, the absence of IGF2BP1 in HCC results in a prolonged half-life and elevated expression levels of HULC.^126^

IGF2BP2 was linked to the m6A modification of GBAP1^89^ and LINC00106,^91^ a process facilitated by METTL3 in HCC (Fig. 4). RNA immunoprecipitation assays demonstrated the specific binding of IGF2BP1 to these two lncRNAs. Suppression of IGF2BP1 resulted in reduced expression of GBAP1 and LINC00106. Inhibition of METTL3 led to a decrease in the association of IGF2BP2 with LINC00106.^89^^,^^91^ Furthermore, Mettl14-mediated m6A methylation was found to enhance the stability of the lncRNA ARHGAP5-AS1, with IGF2BP2 serving as a m6A reader in this context. Knockdown of IGF2BP2 significantly reduced the levels of ARHGAP5-AS1 in HCC cells.^101^ Moreover, IGF2BP2 was observed to increase the stability of LINC01977 in HCC, thereby up-regulating its expression. Notably, LINC01977 has been shown to impede the ubiquitination and degradation of Notch2, thereby promoting the proliferation, metastasis, and invasion of HCC cells. However, the specific molecule that acts as a m6A writer in this process remains unclear.^127^

### YTH domain family

The YTH domain family comprises five members: YTHDF1, YTHDF2, YTHDF3, YTHDC1, and YTHDC2.^128^ YTHDC1 is primarily localized in the nucleus and is involved in the regulation of mRNA splicing and noncoding RNA-mediated gene silencing, impacting RNA nucleation processes.^129^^,^^130^ The other four members are predominantly found in the cytoplasm. YTHDF2 influences RNA stability and facilitates RNA degradation by interacting with the CCR4-NOT complex.^131^ YTHDF1, YTHDF3, and YTHDC2 primarily modulate mRNA translation efficiency.^128^^,^^132^

The YTH domain family plays an important role in regulating the expression of various lncRNAs in HCC (Fig. 4). LINC01273 is linked to sorafenib resistance in HCC cells, and its transcription level is regulated by METTL3-mediated m6A modification. YTHDF2 acts as a m6A reader in this process, controlling the post-transcriptional stability of LINC01273. The knockdown of YTHDF2 markedly increased LINC01273 levels in sorafenib-resistant HCC cells.^98^ The Wnt/β-catenin pathway plays prominent roles in several biological processes including organogenesis, stem cell regeneration, and cell survival.^133^ lncAY overexpression activated the Wnt/β-catenin signaling pathway by up-regulating BMI1 in HCC, enhancing the metastasis and invasion capabilities of HCC cells. Down-regulation of YTHDF2 contributed to a significant increase in the half-life of lncAY transcripts, thereby increasing lncAY expression.^134^ Furthermore, high expression of the lncRNA LEAWBIH in HCC patients was associated with a low survival rate. m6A-modified LEAWBIH interacts with the m6A reader YTHDC1, which is further associated with the H3K9me2 demethylase KDM3B (lysine demethylase 3B) to recruit the CTNNB1 (catenin beta 1) promoter. This interaction leads to H3K9me2 demethylation and CTNNB1 transcriptional activation, promoting the proliferation, migration, and invasion of HCC cells.^100^

YTHDF2 typically facilitates RNA degradation, but under certain conditions, it can also alter RNA splicing. Vulnerability to ferroptosis is closely associated with the development of HCC. lncFAL reduced susceptibility to ferroptosis by interacting with ferroptosis suppressor protein 1 (FSP1) and impeding tripartite motif containing 69 (TRIM69)-mediated polyubiquitination-mediated degradation of FSP1. YTHDF2 plays a direct role in regulating the expression of lncFAL in HCC by binding to prelncFAL, the precursor molecule of lncFAL, and facilitating its conversion into lncFAL, thereby elevating its expression level.^135^

### HuR

HuR, an embryonic lethal abnormal visual (ELAV)-encoded protein, functions to increase the stability of mRNA by binding to target mRNA molecules. This interaction protects the mRNA from degradation by nucleases during transport between the nucleus and cytoplasm, thereby facilitating post-transcriptional gene expression.^136^ In the context of HCC, the lipopolysaccharide produced by intestinal bacteria can increase the expression of METTL14, leading to an increase in the m6A methylation of the lncRNA MIR155HG and subsequent up-regulation of its transcription. By acting as a competing endogenous RNA (ceRNA), MIR155HG promoted the expression of programmed cell death ligand 1 (PD-L1) through the miR-223/signal transducer and activator of transcription 1 (STAT1) signaling pathway, thereby contributing to immune evasion in HCC. HuR functions as a reader protein in the context of m6A methylation and plays a vital role in preserving the post-transcriptional stability of MIR155HG. This stability is essential for sustaining the biological effects of lipopolysaccharide in HCC.^103^

## m5C writers

m5C methyl modification, involving the addition of an active methyl group from the donor to the fifth carbon of the cytosine base within RNA, is a prevalent RNA alteration present in various RNA types.^137^ This modification process is also mediated by three categories of proteins: methyltransferases (writers), demethylases (erasers), and reading proteins (readers).^138^ In studies on HCC, investigations into the impact of m5C modification on lncRNA expression are limited, with only one study highlighting the post-transcriptional regulation of lncRNA H19 by NOP2/Sun-domain family member 2 (NSUN2).^139^ The NSUN family, particularly NSUN2, has been extensively researched as a methyltransferase.^140^ This study revealed that NSUN2 could suppress the proliferation, metastasis, invasion, and angiogenesis of HCC cells. The authors performed comprehensive RNA bisulfite sequencing and RNA sequencing analyses to assess alterations in RNA expression and 5 mC methylation levels after knocking down NSUS2 expression in HCC. This study identified the downstream target lncRNA H19 and revealed that NSUS2 increased H19 m5C methylation, extended H19 half-life in HCC cells, and enhanced H19 stability. Furthermore, researchers have demonstrated that NSUS2-mediated m5C methylation can enhance the interaction between H19 and the oncoprotein G3BP1 (G3BP stress granule assembly factor 1), suggesting that m5C-modified H19 lncRNA might promote tumorigenesis and progression by recruiting the G3BP1 oncoprotein.^139^ In another study, transcriptome-wide 5 mC functional profiling of lncRNAs in six sets of HCC tissues and adjacent noncancerous tissues was carried out. m5C methylation of lncRNAs was more prevalent in HCC tissues than in noncancerous tissues, with m5C frequently resulting in elevated expression of lncRNAs. Kyoto Encyclopedia of Genes and Genomes (KEGG) analysis revealed that these m5C methylation-regulated lncRNAs were involved in various tumor-associated signaling pathways. These findings suggest that m5C methylation of lncRNAs plays a significant role in the progression of HCC.^141^

## miRNAs in the regulation of lncRNA expression

miRNAs are endogenous small noncoding RNA molecules with approximately 22 nucleotides in length.^142^ These small RNA structures can associate with Ago proteins to form RNA-induced silencing complexes (RISCs).^143^ Once the RISC complex is formed, miRNA accurately recognizes the 3′ untranslated region binding site on the target mRNA using nucleotide sequences at the 5′ end. It guides the RISC complex to bind to target mRNAs, leading to the post-transcriptional down-regulation of gene expression.^144^ During the early period, researchers primarily focused on mRNA genes as the targets of miRNAs and established numerous online platforms for predicting miRNA target genes based on the complementary interaction between miRNAs and mRNAs. As miRNA research has progressed, many studies have gradually revealed that, in addition to mRNA genes, noncoding RNAs can also be regulated by miRNAs. The concept of ceRNA has been proposed, suggesting that lncRNAs and other noncoding RNAs contain miRNA response elements and can competitively bind to the same miRNA species as coding gene mRNAs. This interaction results in the formation of a complex regulatory network involving lncRNAs, miRNAs, and mRNAs.^145^

A previous study indicated that miRNAs play a role in regulating the post-transcriptional levels of lncRNAs in HCC. In 2015, two independent research teams simultaneously investigated the post-transcriptional regulation of the lncRNA HOTTIP (HOXA distal transcript antisense RNA) by miRNAs in HCC. Ge et al utilized miRCode software to predict potential miRNAs that could regulate the lncRNA HOTTIP. Through luciferase reporter gene experiments, they identified miR-192 and miR-204 as direct regulators of HOTTIP, inhibiting the proliferation of HCC cells through the miR192/204-lncRNA HOTTIP-GLS1 (glutaminase 1) signaling axis.^146^ Similarly, Tsang et al highlighted the post-transcriptional regulation of HOTTIP by miRNAs in HCC. They demonstrated that miR-125b directly targeted HOTTIP and that the overexpression of miR-125b in HCC cell lines significantly decreased HOTTIP expression.^147^ Furthermore, Zhao et al reported that in the tumor tissues of HCC patients, miRNA-124 levels were reduced and lncRNA-UCA1 (urothelial carcinoembryonic antigen 1) levels were increased compared with those in adjacent nontumor tissues. Low miRNA-124 and high lncRNA-UCA1 levels were strongly associated with unfavorable survival outcomes. Overexpression of miR-124 in HCC cells led to a decrease in UCA1 expression and a notable inhibition of cell proliferation, metastasis, and invasion.^148^ In a study conducted by Deng et al in 2021, the authors reported that miR-135a was hyper-expressed and that the lncRNA TONSL-AS1 was hypo-expressed in HCC tissues. The expression level of lncRNAs has a positive impact on the prognosis of patients with HCC. The overexpression of miR-135a in HCC cells could reduce the expression of lncRNAs and promote the proliferation of HCC cells, while the overexpression of lncRNAs had no such effect on the expression of miR-135a. The authors concluded that miR-135a could facilitate the progression of HCC cells by suppressing the expression of the lncRNA TONSL-AS1.^149^ A recent study highlighted the inhibitory effect of miR-155 on lncRNA PCTA29 (prostate cancer-associated transcript 29) in HCC, which resulted in the suppression of the metastasis and invasion capabilities of HCC cells. Additionally, the expression level of PCTA29 was significantly linked to the prognosis of HCC patients.^150^ These findings underscore the limited research on the post-transcriptional regulation of lncRNAs in HCC, with only two studies explicitly confirming the targeting relationship between miRNAs and lncRNAs through luciferase reporter gene experiments.^146^^,^^147^ Other studies have established a negative correlation between miRNA and lncRNA expression levels without definitively establishing a direct targeting mechanism. As suggested by Braconi et al, it is plausible that intermediary molecules exist between miRNAs and lncRNAs, influencing the regulation of lncRNA transcription.^42^

## Conclusion and future perspectives

This review offers a comprehensive analysis of the complex regulatory network of epigenetic modifications on lncRNAs in HCC, highlighting the impact at both transcriptional and post-transcriptional levels. Epigenetic modifications, including DNA methylation, histone modifications, RNA modifications, and miRNA regulation, play a significant role in the regulation of lncRNA expression in HCC. While current research has shed light on RNA modifications, particularly the m6A methylation, there is a need for more in-depth investigations into the roles of DNA methylation, histone modifications, and miRNAs in this context.

Understanding the intricate mechanisms by which these epigenetic marks regulate lncRNAs is essential for deciphering the pathogenesis of HCC. The interplay between epigenetic modifications and lncRNAs suggests the potential for these molecules to serve as sensitive biomarkers for HCC diagnosis and prognosis. Furthermore, the dysregulation of specific lncRNAs due to epigenetic alterations could be harnessed as therapeutic targets, leading to the development of new treatment strategies that modulate their expression or function.

In conclusion, the epigenetic regulation of lncRNAs in HCC is a multifaceted and dynamic area that merits further exploration. Elucidating the complex interactions between epigenetic modifications and lncRNA biology will enhance our comprehension of HCC and potentially lead to the development of innovative diagnostic and therapeutic modalities.

## Funding

This work was supported by the 10.13039/501100001809National Natural Science Foundation of China (No. U23A20487, 82001874, 82102105), the Zhejiang Engineering Research Center of Cognitive Healthcare (China) (No. 2017E10011), the 10.13039/501100004731Natural Science Foundation of Zhejiang Province, China (No. LQ22H160017), the Zhejiang Province Science and Technology Plan Project (China) (No. 2022C03134), and the Science and Technology Innovation 2030 Plan Project of China (No. 2022ZD0160703).

## CRediT authorship contribution statement

**Zhaoqi Shi:** Writing – original draft, Visualization, Methodology, Conceptualization. **Shengxi Jin:** Visualization, Methodology. **Xiaolong Liu:** Visualization, Supervision. **Mengting Jiang:** Methodology. **Yifeng Fang:** Visualization. **Parikshit Asutosh Khadaroo:** Writing – review & editing. **Hui Lin:** Supervision. **Xiaoxiao Fan:** Writing – review & editing, Supervision, Methodology, Conceptualization.

## Conflict of interests

The authors declared no conflict of interests.
